# Single-test syphilis serology: A case of not seeing the forest for the trees

**DOI:** 10.1371/journal.pone.0303253

**Published:** 2024-05-09

**Authors:** Ethan Mabvuto Zulu, Julie M. Herlihy, Cassandra R. Duffy, Lawrence Mwananyanda, Roma Chilengi, Leah Forman, Tim Heeren, Christopher J. Gill, Roy Chavuma, Barbara Payne-Lohman, Donald M. Thea

**Affiliations:** 1 Right to Care Zambia, Lusaka, Zambia; 2 Department of Pediatrics, Boston Medical Center, Boston University, Chobanian & Avedisian School of Medicine, Boston, Massachusetts, United States of America; 3 Department of Global Health, Boston University School of Public Health, Boston, Massachusetts, United States of America; 4 Department of Obstetrics & Gynecology, Division of Maternal-Fetal Medicine, Beth Israel Deaconess Medical Center, Harvard Medical School, Boston, Massachusetts, United States of America; 5 Centre for Infectious Disease Research in Zambia, Lusaka, Zambia; 6 Biostatistics and Epidemiology Data Analytics Center, Boston University School of Public Health, Boston, Massachusetts, United States of America; 7 Department of Biostatistics, Boston University School of Public Health, Boston, Massachusetts, United States of America; 8 Institute for Immunology and Informatics, University of Rhode Island, Kingston, Rhode Island, United States of America; Universita degli Studi di Bologna Scuola di Medicina e Chirurgia, ITALY

## Abstract

**Introduction:**

There have been few empirical studies for diagnostic test accuracy of syphilis using a sequence of rapid tests in populations with low prevalence of syphilis such as pregnant women. This analysis describes syphilis test positivity frequency among pregnant women at an antenatal clinic in Zambia using a reverse-sequence testing algorithm for antenatal syphilis screening.

**Methods:**

Between August 2019 and May 2023, we recruited 1510 pregnant women from a peri-urban hospital in Lusaka, Zambia. HIV positive and HIV negative women were enrolled in a 1:1 ratio. Blood collected at recruitment from the pregnant mothers was tested on-site for syphilis using a rapid treponemal test. Samples that tested positive were further tested at a different laboratory, with rapid plasma reagin using archived plasma.

**Results:**

Of the total 1,421 sera samples which were screened with a rapid treponemal test, 127 (8.9%) were positive and 1,294 (91.1%) were negative. Sufficient additional samples were available to perform RPR testing on 114 of the 127 (89.8%) RDT positive specimens. Thirty-one (27.2%) of these 114 were reactive by RPR and 83 (72.8%) were negative, resulting in a syphilis overtreatment rate of 3 fold (i.e, 84/114). Insufficient sample or test kit availability prevented any testing for the remaining 89 (5.9%) participants.

**Conclusion:**

Use of only treponemal tests in low prevalence populations, like pregnant women, subjects individuals with non-active syphilis to the costs and possible risks of overtreatment. The use of the dual treponemal and non-treponemal tests would minimize this risk at some additional cost.

## Background

Syphilis, a sexually transmitted disease caused by bacterium *Treponema pallidum*, remains the second leading global cause of stillbirths. Untreated maternal syphilis result in congenital syphilis in 80% of the cases leading to significant adverse birth outcomes in 52% of pregnant women [1]. In Zambia, data about case rates of congenital syphilis at subnational and national level are limited. However, the prevalence of syphilis among pregnant women in Zambia during the 2018 to 2019 period was reported to be 5.6% [2].

Syphilis screening of all pregnant women is part of the basic antenatal care (ANC) package recommended by the World Health Organisation (WHO) as a cost-efficient method to reduce the incidence of congenital syphilis [3]. In recent years, there has been a growing trend towards using point-of-care rapid tests for syphilis, including rapid syphilis test (RST) and rapid plasma reagin (RPR) to allow for same-visit treatment [4]. While there is evidence of single-test strategies amongst the general population, there have been few empirical studies for diagnostic test accuracy using a sequence of tests in populations with low prevalence of syphilis such as pregnant women [5]. This creates major challenges in syphilis surveillance reporting and management among laboratories, clinicians and public health practitioners, such as overestimation of syphilis seroprevalence and overtreatment of syphilis since a positive treponemal test result alone can persist over a lifetime.

To improve pregnancy and birth outcomes, the Zambia Ministry of Health recommends syphilis testing to be conducted in every trimester of pregnancy [6]. The most common point-of-care tests for syphilis used in Zambia can be classified into two types: treponemal and non-treponemal tests [7]. Treponemal tests include Enzyme immunoassay (EIA), IgM-Enzyme Immunoassay, Fluorescent Treponemal Antibody Absorption (FTA-abs), Treponemal Pallidum Hemagglutination Assay (TPHA) and Treponemal Pallidum Particle Agglutination (TPPA). Conversely, the non-treponemal tests, which are not specific to syphilis, include the RPR and the Venereal Disease Research Laboratory (VDRL) [7]. Recently, there is considerable use of dual rapid HIV and treponemal syphilis screening tests in hospitals. While the RPR test was used in the past for initial screening purposes, the advent of automation and treponemal rapid tests has led to a rapid increase of new treponemal tests on the market [8]. Consequently, the Zambia Ministry of Health has adopted the “reverse algorithm for syphilis” which involves an initial treponemal antibody screening immunoassay test, followed by confirmation with non-treponemal antibody testing (RPR), if available.

While the screening guidelines advocate the “reverse algorithm for syphilis” diagnosis, the health system in Zambia still faces several challenges in policy implementation. One such challenge is the erratic supply of testing kits and essential medicines [7,9]. In accordance with WHO guidelines for syphilis screening and treatment among pregnant women, it is common for health facilities to perform single serology syphilis tests and provide same-day treatment based on positive results of the rapid test only, without confirmatory testing [3]. This may result in overtreatment of pregnant women without *active* syphilis, defined as positivity on both a treponemal and nontreponemal test [3,10,11].

A recent meta-analysis of 44 studies showed a 2.87% pooled prevalence of syphilis among pregnant women in sub-Sahara Africa [12]. However, nearly all of the included studies used results from either a single test strategy or the traditional approach, except for one study by Taylor *et al* which reported on syphilis seropositivity using reverse-sequence testing algorithm [13].

This manuscript describes syphilis positivity results among pregnant women at an antenatal clinical located in peri-urban Lusaka, Zambia using a reverse-sequence testing algorithm for antenatal syphilis screening (treponemal testing followed by nontreponemal testing for confirmation) with the aims to: estimate active syphilis cases in a pregnant population; predict occurrence of possible overtreatment among pregnant women testing positive with a single treponemal test and balance antimicrobial stewardship with the risk of pregnant women at high risk of delivering infants with congenital syphilis.

## Methods

### Population

We tested blood of pregnant women at antenatal first booking who were enrolled into a prospective cohort study investigating whether HIV-exposed but uninfected infants (HEU) are at increased risk of mortality and morbidity compared to their HIV- unexposed uninfected (HUU) counterparts. The study, the Zambia Infant Cohort Study, was approved by both the University of Zambia Biomedical Research Ethics and Boston University Institutional Review Board. Between August 2019 and May 2023, we recruited 1510 participants from a peri-urban hospital in Lusaka, the capital city of Zambia. Both HIV positive and HIV negative women were enrolled in a 1:1 ratio.

Following an explanation of what study participation entailed, the participant was offered an opportunity to have all of their questions answered before consenting to participation in the study. Written informed consent was obtained from every participant. For minors, written informed consent was obtained from the parents or guardians. A copy of the consent form was given to the participant and the signed original was maintained with the study records.

### Laboratory testing

Blood collected at recruitment was initially tested on-site for syphilis using a rapid treponemal test following the manufacturer’s instruction. The syphilis rapid test (syphilis strip test manufactured by BHAT, Bengaluru City, India) uses chromatographic immunoassay for the qualitative detection of antibodies (either IgG or IgM) to *Treponema pallidum* in serum or plasma. Samples that tested positive were further tested at a different laboratory, the CLIA approved Center for Infectious Diseases Research Zambia with rapid plasma reagin (Becton Dickson Macro-Vue^TM^ RPR Card Tests) using archived plasma. A titre of 1:4 or higher was considered reactive. No further testing was done for those samples that initially tested negative with the initial rapid treponemal test. Active infection was defined as (RDT + / RPRT +). Past infection was defined as (RDT + / RPRT -).

### Statistical analyses

We compared baseline maternal factors and syphilis infection between HIV-infected and HIV-uninfected pregnant mothers using *χ *^*2*^ tests or Fisher’s exact tests (where cell frequencies where small) for categorical factors and Wilcoxon Rank Sum tests for continuous variable.

## Results

Table 1 shows baseline characteristics of the women included in the syphilis testing. Both groups were of comparable socio-economic status, as shown in all five socioeconomic status quintiles. The mean age of participants was 28, with women living with HIV (WLHIV) being slightly older (mean of 29 years vs. mean of 26 years for the uninfected, p < .0001), owing likely to their older age and longer period of sexual activity. WLHIV were more likely to have active syphilis infection (4.1% vs. 1.2%, p = 0.0007) and past syphilis infection compared to HIV-uninfected participants (7.8% vs. 2.8%, p <0.0001). The majority of the WLWHIV had been on ART for more than 6 months (525/731, 71.4%). Alcohol use in pregnancy was more common among WLHIV than HIV-uninfected (15.1% v 7.1%, p<0.0001).

**Table 1:** Demographic characteristics of the 1421 pregnant women in the Zambia Infant Cohort Study who were screened for syphilis.

			HIV Infection Status		
Variable	Response	Overall (n = 1421)	No (n = 686)	Yes (n = 735)	p-value^*^
Syphilis: never positive / active infection / past infection	Never positive (negative RDT)	1294 (91.1%)	655 (95.5%)	639 (86.9%)	< .0001
	Active infection (RDT + / RPRT +)	38 (2.7%)	8 (1.2%)	30 (4.1%)	
	Past infection (RDT + / RPRT -)	76 (5.3%)	19 (2.8%)	57 (7.8%)	
	Unknown status (RDT + / RPRT missing)	13 (0.9%)	4 (0.6%)	9 (1.2%)	
Age in years	Mean (Std Dev)	28 (6)	26 (6)	29 (6)	< .0001
ART initiation timing	HIV negative	‐‐	‐‐	4 (0.5%)	‐‐
	ART naïve	‐‐	‐‐	128 (17.4%)	
	<6 months	‐‐	‐‐	78 (10.6%)	
	> = 6 months	‐‐	‐‐	525 (71.4%)	
Alcohol during current pregnancy	No	1261 (88.7%)	637 (92.9%)	624 (84.9%)	< .0001
	Yes	160 (11.3%)	49 (7.1%)	111 (15.1%)	
SES (Urban) Quintile	1st quintile	52 (3.7%)	25 (3.6%)	27 (3.7%)	0.3783
	2nd quintile	292 (20.7%)	147 (21.5%)	145 (19.9%)	
	3rd quintile	573 (40.6%)	260 (38.0%)	313 (43.0%)	
	4th quintile	381 (27.0%)	197 (28.8%)	184 (25.3%)	
	5th quintile	115 (8.1%)	56 (8.2%)	59 (8.1%)	

Abbreviations: RPRT, rapid plasma reagin test; ART, antiretroviral therapy; SES, socio-economic status.

* Unadjusted p-value from Pearson χ^2^ test.

**Fig 1:**
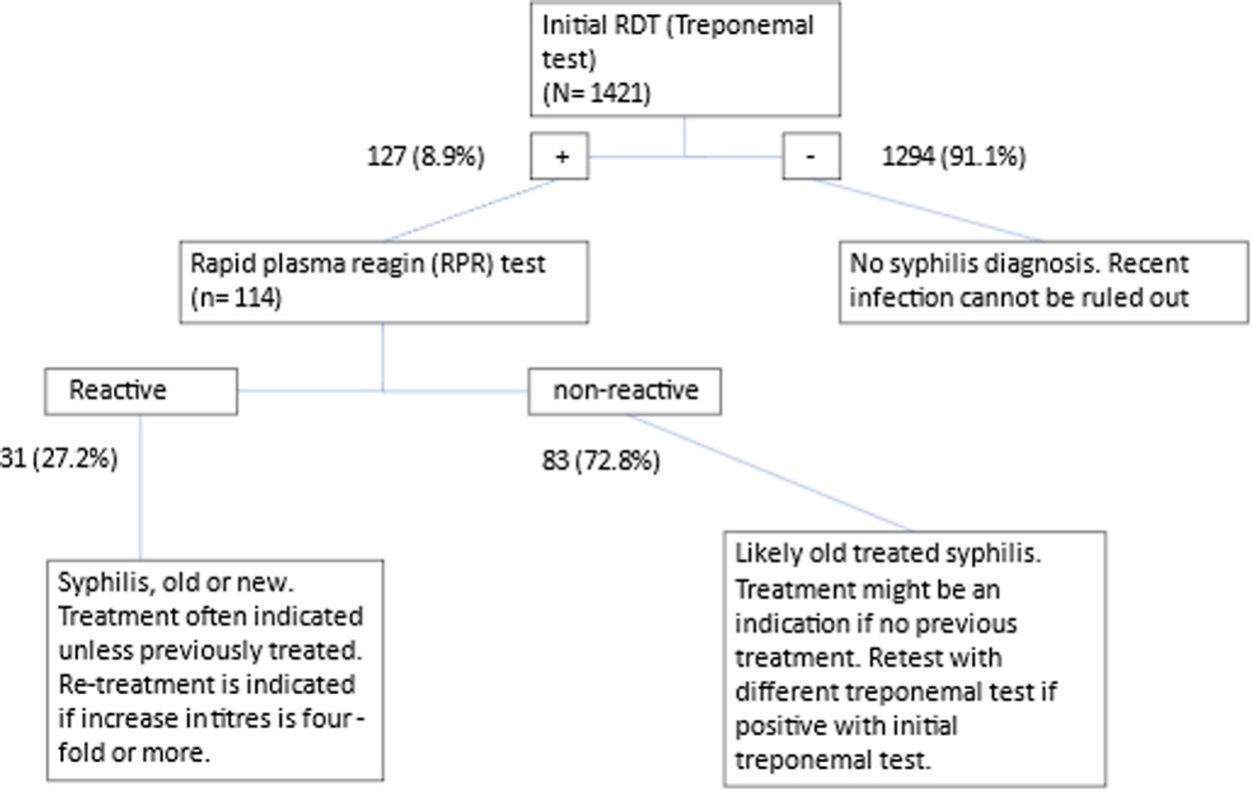
Flow chart of samples and testing. Abbreviations: RDT = rapid diagnostic test.

Syphilis testing was performed at enrollment on 735 HIV-infected and 686 HIV-uninfected pregnant women (N = 1421), as depicted in Fig 1. Of the total 1,421 sera which were screened with a rapid treponemal test, 127 (8.9%) were positive and 1,294 (91.1%) were negative. All women with a positive rapid test were offered treatment for syphilis with three doses of benzathine penicillin. Treatment with a single dose of penicillin is not standard practice because of lack of reagents to monitor titres. Sufficient additional sample was available to perform RPR testing on 114 of the 127 (89.8%) RDT positive specimens. Thirty-one (27.2%) of these 114 were reactive by RPR and 83 (72.8%) were non-reactive resulting in an overtreatment rate of 84/114. Thus, nearly 3-fold more pregnant women without active syphilis would be recommended for treatment based on results of rapid testing alone for each PW with active syphilis confirmed by RPR. Insufficient sample or test kit availability prevented any testing for the remaining 89 (5.9%) participants.

## Discussion

Using only treponemal testing resulted in a higher overtreatment rate when compared to using a reverse testing algorithm which deploys both treponemal and non-treponemal tests. This observed difference could be attributed to treponemal tests’ greater sensitivity and inability to distinguish past or previously treated syphilis infection from active infection [11]. Treating all treponemal positive-tested pregnant women who are yet unconfirmed by nontreponemal testing will result in over treatment. Current practice is premised on the notion that the risk associated with untreated maternal syphilis and risk of congenital syphilis significantly outweighs the risk of overtreatment. Congenital syphilis has dire consequences for infected infants and an aggressive treatment strategy may be a reflection of that risk. However, the implementation of a reverse testing algorithm for syphilis may reduce overtreatment and unnecessary exposure to antibiotics, while still mitigating the risk of vertical transmission with more precision. Recent research suggests that antibiotic treatment during pregnancy may alter infant gut microbiota and increases risk for sepsis and necrotizing enterocolitis (NEC), particularly in premature infants [14]. To date, *Treponema pallidum* has not shown any documented resistance to penicillin but given the global emergence of widespread antimicrobial resistance, antibiotic stewardship remains a priority.

While the Zambia national policy recommends the reverse algorithm for syphilis screening, we witnessed implementation deficits at our study site which was subject to hospital resources–and shortages–for syphilis testing. On several occasions, there were stock-outs of treponemal tests, and confirmation at the hospital associated laboratory testing with RPR largely did not occur due to lack of reagents. In resource-constrained settings like ours, frontline workers were left to report only unconfirmed syphilis results from a treponemal test and only when RDT kits were available. This has serious implications on the accuracy of syphilis surveillance in that community and ultimately in the national statistics. For example, according to 2021 WHO global health observatory data, only 44% of women who accessed ANC services received a syphilis test and of the 3.4% who tested positive, it is not clear how many were treated [15]. Worse still, periodic stock-outs of benzathine penicillin G are often observed in Zambian clinics, made worse recently by the general disruptions of supply chains created by the COVID-19 pandemic [9]. National and global shortages of benzathine penicillin related to production and procurement also call for targeted use [16].

In designing healthcare policy, a major factor is the cost-effectiveness of the testing strategy. Unfortunately, it is extremely difficult to make meaningful statements about which algorithm is cost-effective without either knowing the financial threshold for the local healthcare system or true prevalence of syphilis. Only a few studies have attempted to undertake the research on the cost-effectiveness of serology syphilis testing algorithms in low middle-income countries. In a study investigating cost-effectiveness of several algorithms, Fern et al. found the use of a single rapid treponemal diagnostic test was the most cost-effective diagnostic approach in Peru, Tanzania and Zambia, while mass treatment was the most-cost effective approach in the high prevalence settings [17]. Further, the reverse algorithm for syphilis screening performed better in identifying true cases while reducing overtreatment but was not as cost-effective as a one-step approach [17]. However, one concern with these assessments is that they did not capture the costs of treating the partner of an infected mother, the exposed infant or indeed repeated treponemal tests and treatment for those who continued to be positive in subsequent pregnancies. Importantly, none of these studies considered the cost of overtreatment and antimicrobial resistance at population level.

On the contrary, a study by Owusu-Edusei et al, found the treponemal-only algorithm to be less cost-effective compared to the 2-step algorithms in low prevalence syphilis settings [18]. While data in this study was from a high-income country, it still illustrates the possibility that public clinics with higher rates of previously treated syphilis may be incurring higher costs by using a treponemal-only algorithm because of the approach’s inability to distinguish active from prior infections.

In light of emerging evidence of the potential risk of sepsis and NEC due to antibiotic exposure, a new cost-effectiveness model will need to include the cost of treating sequelae of antibiotic exposure [14,19]. Ultimately, this calls for a strengthened syphilis-testing strategy that would balance the risk of missing infants at high risk of acquiring congenital syphilis with the adverse outcomes of antibiotic overuse. There is now in use a rapid treponemal/nontreponemal test that can detect positivity on both of these serologies in a single test cartridge. This test is currently under review for performance in the United States and would allow for identification of point-of-care active syphilis when used in antenatal care settings thus facilitating appropriate treatment [11].

Finally, and though not shown in this paper, when exploring the data on whether syphilis was a potential confounder or mediator of the association between HIV exposure and adverse birth outcomes, it was not surprising that the results of our analyses differed depending on which algorithm for syphilis screening (treponemal-only algorithms or reverse algorithm) was used. For example, the association between HIV exposure and syphilis on birth outcomes was statistically significant when results from the treponemal-only algorithm were used. However, this association with HIV exposure was attenuated and no longer statistically significant when using results from the reverse algorithm for syphilis diagnosis. This provides a cautionary tale to clinicians, epidemiologists, ministries of health, partners and researchers of how to interpret syphilis results.

## Conclusion

Use of only treponemal tests in low prevalence populations, like pregnant women, introduces the problem of identifying non-active syphilis resulting in subsequent overtreatment. Treponemal EIAs are cheap and are becoming increasingly available, but it is important to remember that confirmation of results by RPR is equally necessary for monitoring syphilis treatment. The use of the dual treponemal and non-treponemal tests would address the problem of overtreatment and promote antibiotics stewardship. Thus, there is need for consistent supply of testing reagents to ensure universal syphilis screening of all pregnant women. More importantly, further studies are needed to establish which syphilis algorithm offers both cost-effectiveness and health benefits in the Zambian context. With growing evidence of association of antibiotic use and potentially harmful changes in gut microbiota, future cost-effectiveness studies will have to incorporate costs from antibiotic overuse.

## Supporting information

S1 File Syphilis results by year. (DOCX)

S2 File Syphilis dataset. (CSV)
