# Supplementary material for: Single-test syphilis serology: A case of not seeing the forest for the trees
Source: PLoS One. 2024 May 9;19(5):e0303253. doi: 10.1371/journal.pone.0303253 (PMC11081208; doi:10.1371/journal.pone.0303253)
Supplement: S1 File — (DOCX) [file pone.0303253.s001.docx]

**S1 Table.** **Syphilis results by year**

**Date run: February 22, 2024**

|  | | Year | | | |
| --- | --- | --- | --- | --- | --- |
| Question | Response | 2019 | 2020 | 2021 | 2022 |
| Total N |  | 311 | 375 | 572 | 163 |
| Syphilis RDT result | 1: Reactive | 11 (3.5%) | 43 (11.5%) | 49 (8.6%) | 24 (14.7%) |
|  | 2: Non-Reactive | 300 (96.5%) | 332 (88.5%) | 523 (91.4%) | 139 (85.3%) |
| RPRT 1:4 or higher | 0: No | 5 (50.0%) | 27 (75.0%) | 35 (76.1%) | 16 (72.7%) |
|  | 1: Yes | 5 (50.0%) | 9 (25.0%) | 11 (23.9%) | 6 (27.3%) |
